# Few-layer black phosphorus enables nitrogen fixation under ambient conditions†

**DOI:** 10.1039/d3ra07331a

**Published:** 2024-02-05

**Authors:** Francisco Garnes-Portolés, Vicent Lloret, José Alejandro Vidal-Moya, Mario Löffler, Karl J. J. Mayrhofer, Jose Pedro Cerón-Carrasco, Gonzalo Abellán, Antonio Leyva-Pérez

**Affiliations:** a Instituto de Tecnología Química, Universitat Politècnica de València-Consejo Superior de Investigaciones Científicas Avda. de los Naranjos s/n 46022 Valencia Spain anleyva@itq.upv.es +34 9638 77809 +34 963877800; b Department of Chemistry and Pharmacy, Joint Institute of Advanced Materials and Processes (ZMP), Friedrich-Alexander-Universität Erlangen-Nürnberg (FAU) Henkestrasse 42, 91054 Erlangen and Dr.-Mack Strasse 81 90762 Fürth Germany +49 91165078-65015 +49 91165078-65031; c Helmholtz-Institute Erlangen-Nürnberg for Renewable Energy (IEK-11), Forschungszentrum Jülich GmbH Cauerstr. 1 91058 Erlangen Germany; d Department of Chemical and Biological Engineering, Friedrich-Alexander University Erlangen-Nürnberg Cauerstr. 1 91058 Erlangen Germany; e Centro Universitario de la Defensa, Academia General del Aire, Universidad Politécnica de Cartagena C/ Coronel López Peña S/N, Santiago de La Ribera 30720 Murcia Spain; f Instituto de Ciencia Molecular (ICMol), Universidad de Valencia Catedrático José Beltrán 2 46980 Paterna Valencia Spain gonzalo.abellan@uv.es

## Abstract

Nitrogen (N_2_) fixation is a key reaction in biological and industrial chemistry, which does not occur spontaneously under ambient conditions but often depends on very specific catalysts and harsh reaction processes. Here we show that exposing exfoliated black phosphorus to the open air triggers, concomitantly, the oxidation of the two-dimensional (2D) material and the fixation of up to 100 parts per million (0.01%) of N_2_ on the surface. The fixation also occurs in pristine non-exfoliated material. Besides, other allotropic forms of phosphorus, like red P, also fixes N_2_ during ambient oxidation, suggesting that the N_2_ fixation process is intrinsic with phosphorus oxidation and does not depend on the chemical structure or the dimensionality of the solid. Despite the low amounts of N_2_ fixed, this serendipitous discovery could have fundamental implications on the chemistry and environmental stability of phosphorous and the design of related catalysts for N_2_ fixation.

## Abstract 2

Nitrogen fixation occurs spontaneously during the oxidation of elemental phosphorus under ambient conditions, although in low extent (<0.01 wt%). However, this discovery could have implications in the chemistry of these two basic elements.

## Graphical abstract

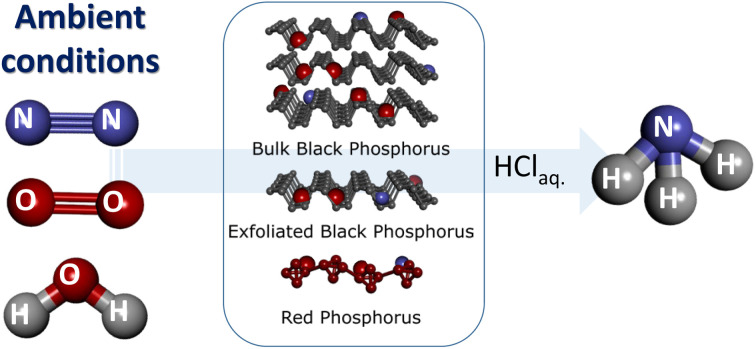

## 1 Introduction

The transformation of unreactive N_2_, present in the air, into useful forms for mankind was a research pursuit during decades, crystallized in the high energy-consuming Haber-Bosch process, which still constitutes the motor drive of our economy and way of life. However, Nature is able to fix nitrogen under ambient conditions with nitrogenase enzymes, and many studies to mimic this simple catalytic system are still on-going.^1^ Nitrogenase enzymes contain Fe as the catalytic active metal, thus it is not surprising that many of the synthetic catalysts reported to date are based on Fe or related transition metals.^2–6^ Nevertheless, a new line of work based on metal-free thermal, photo- and electro-catalysts has emerged during the last years, which avoids the need of a single metal site but use extended heteroatomic surfaces to adsorb and inject electrons on the highly unreactive N≡N bond.^7^ Some recent theoretical and experimental works reveal that pristine two-dimensional (2D) few-layer black phosphorous (FL-BP)^8–10^ as well as doped with other heteroatoms such as B, N or O,^11^ or metals such as Fe,^12^ is among the more effective materials for this transformation, allowing photo-, and also, electro-catalysis for N_2_ fixation at room temperature.^13^ However, a recent study based on spin-polarized density functional theory (DFT) has shown that, in principle, pristine FL-BP may activate and fix N_2_ without the assistance of any extrinsic driving force.^12^

**Scheme 1:**
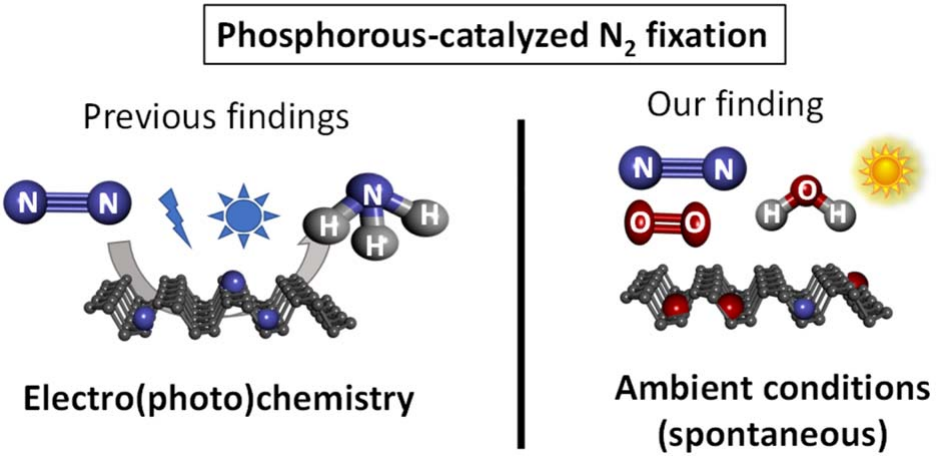
Previous work on phosphorous-catalyzed N_2_ fixation and the spontaneous fixation on elemental phosphorous under ambient conditions reported herein. Ammonia is obtained after hydrolysis.

As far as we know, the spontaneous fixation of N_2_ under ambient conditions without any external help has not been reported for any elemental material, since the high energy associated to the breaking of the N≡N bond (942 kJ mol^−1^) hampers any process to overcome such a high energy. Here we show that the ambient oxidation of solid FL-BP, by simply leaving a sample of the 2D material in a bench at the open air, produces the spontaneous fixation of significant amounts of N_2_, as shown in Scheme 1. The energy required for this serendipitously found process comes from the concomitant oxidation of the sample, thus leaving a final N–P–O composed material. After hydrolysis, NH_3_ is formed. It is worthy commenting that the study reported here is not aimed at producing NH_3_, but at describing the spontaneous reactivity of ambient N_2_ with different elemental phosphorous forms.

## 2 Results and discussion

We first tested the fixation of N_2_ with thin-layered BP nanosheets. The samples were achieved in three steps: (1) sonication horn in NMP, (2) centrifugation, and (3) solvent transfer to THF. To check if the amount of active sites depends on the solid surface, we then tested pristine BP stones without any exfoliation process and red phosphorus (red P), and both solid showed this effect, which suggests that any allotropic form of elemental phosphorus could fix nitrogen during oxidation.

**Fig. 1:**
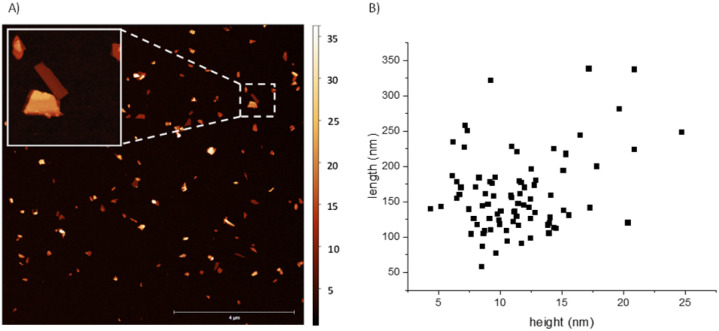
(A) 10 × 10 μm AFM image of the exfoliated BP nanosheets after centrifugation and solvent transfer to THF. Bar represents 4 μm. Insert shows a 1 × 1 μm zoom in, of a 19 nm (bottom) and 7 nm (top) thick flakes selected in the area with the dashed line square. (B) Manually extracted statistics of length and height in nm of the exfoliated material calculated with 80 flakes.

The detailed exfoliation of BP to obtain thin and small nanosheets has been optimized in our laboratories by changing four different parameters that affect the exfoliation procedure, namely: the starting concentration, the applied sonication amplitude, the time, and the centrifugation force. This has led to a standardized and reproducible process to obtain BP nanosheets with *ca.* 100 to 700 nm length and a mean height of *ca.* 15 nm. Thus, samples prepared with 36 mg of starting material in 15 mL of NMP, sonicated at 80%, and centrifuged at 1753 g for two hours led to an estimated 20 μg mL^−1^ concentration. It is important to stress that this procedure can be taken as a model to exfoliate BP, but we recommend optimizing the exfoliating protocol for each laboratory set, since we have observed that conditions such as the type of sonotrode tip, centrifuge, exfoliating vials, and temperature strongly influence the size and concentration of the final dispersions.^14,15^ As previously mentioned, after the exfoliation in NMP and the centrifugation processes, a solvent transfer step – also developed in our laboratories – is implemented to change the solvent of the dispersion to THF (process described in the ESI†). The last is easier to handle due to its slower viscosity, also facilitating the cleaning step of drop-casted dispersions on substrates. Fig. 1 shows a 10 × 10 μm AFM image with the height and length statistics extracted manually. For this sample, considering a total of 84 flakes, an average height of 11.4 ± 3.9 nm and length varying between 50 and 350 nm with a mean 161.8 ± 56.0 nm was obtained, in excellent agreement with the previously mentioned values for this sort of liquid phase exfoliation (LPE) BP samples. An enlarged AFM image and four zooms are shown in Fig. S1.†

**Fig. 2:**
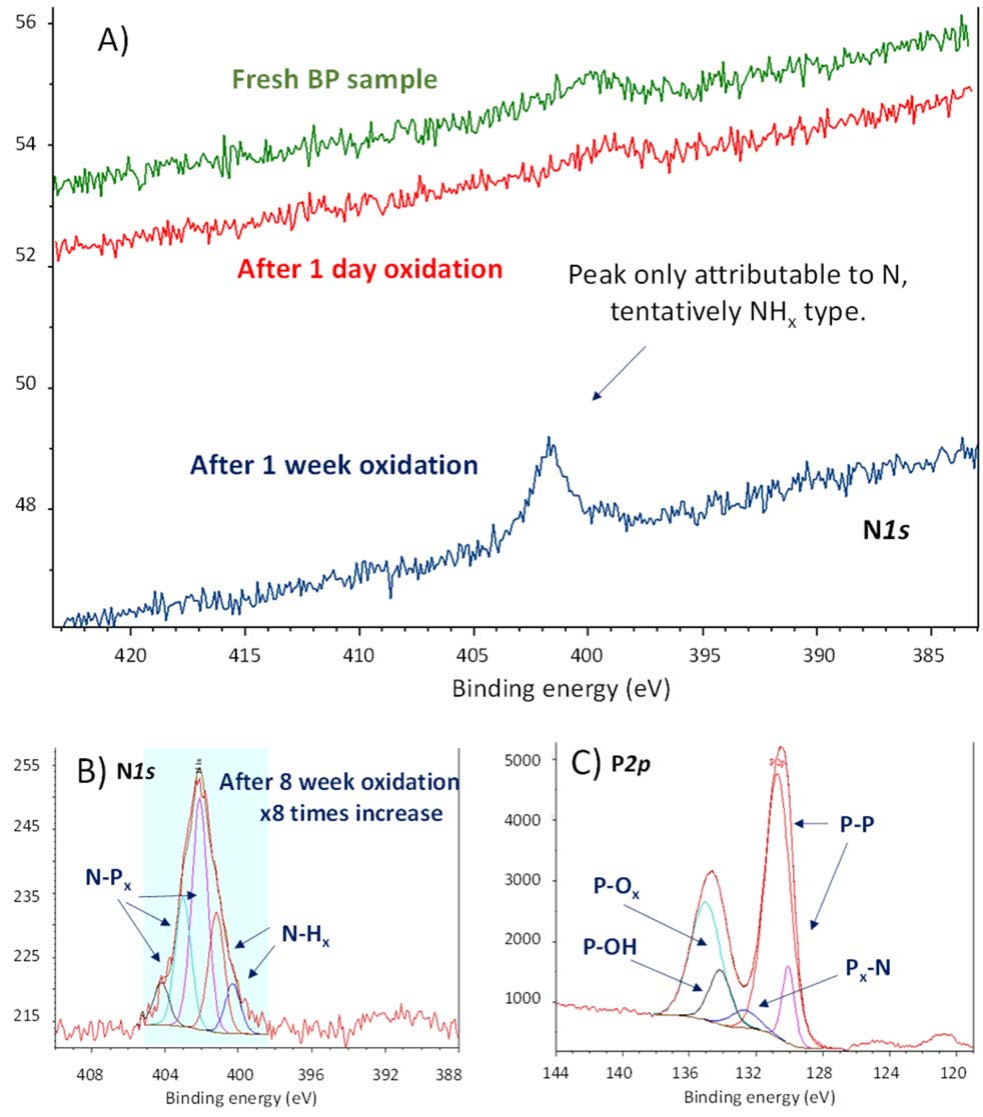
(A) N1s X-ray photoelectron spectra (XPS) of fresh FL-BP (green line), and left under ambient conditions for 1 day (red line) and 1 week (blue line) respectively, managed and recorded at HIERN laboratory. (B) Deconvoluted high-resolution N1s XPS of FL-BP left under ambient conditions for 8 weeks, managed and recorded at ITQ-UPV/CSIC laboratory. (C) Deconvoluted high-resolution P2p XPS of the previous sample (B).

These materials were left to the open air in a bench of one of the author's laboratory (HIERN, Germany), and their evolution was followed by X-ray photoelectron spectroscopy (XPS). Amongst all the characterization techniques used for BP oxidation tracking so far (Raman, UV-Vis, photoluminescence, XRD, *etc.*), XPS is probably the most promising one to understand its reactivity, since the rest of the techniques will show how the material decomposes without significant information on the chemical structure. Fig. 2A shows that a new signal centered at 402.0 eV emerges in the X-ray photoelectron spectrum (XPS) of FL-BP when a sample is left on the bench for one week. This signal could only be assigned to N species on the surface, in particular to different NP_*x*_ and NH_*x*_ species after deconvolution,^16^ shown in Fig. 2B.

In order to see if the N_2_ fixation is only happening with the exfoliated material, we also tested pristine BP, without the exfoliation step. The experiment was repeated in a different laboratory (ITQ-UPV/CSIC, Spain), and the result shows the same trend (Fig. S2†). Indeed, a literature search shows that the N1s XPS signal at 402 eV appears in all the XPS survey graphs reported for aerobically oxidized samples of FL-BP and phosphorene, at least those we could find (for selected examples see Fig. S3–S5†).^17–19^ However, this N signal does not appear when the oxidation is performed with other agents different to air, *i.e.* metal salts and water (Fig. S6†),^20^ which suggests that N incorporation into FL-BP proceeds from ambient N_2_. The relative intensity of the N peaks increases with time when exposed to ambient conditions (Fig. S7†), with respect to the total P amount, and correlates well with the increase in P–O_*x*_ signals in XPS, as shown in Fig. 2C. Indeed, the deconvolution of these P2p XPS signals shows the appearance of a new peak at 132.2 eV, which can be assigned to P–N bonds.^16^ The other deconvoluted peaks can be attributed to the original P–P bonds in FL-BP and the new oxidized P–O bonds, respectively, according to reported values and the corresponding O1s XPS spectrum (Fig. S8†).^21,22^ Notice that the P2p XPS signal of a barely oxidized sample is somewhat different [see for instance Fig. S6 (right) and S7 (top, middle graph)†]. These results indicate the incorporation of N atoms in the FL-BP structure concomitantly with the ambient oxidation of the 2D material, and this process seems to be general and previously ignored in the literature.

Spin-polarized density functional theory (DFT) studies^12^ have indicated that the presence of doping Fe atoms anchored on the channel of P catalyzes the N_2_ reduction reaction. Thus, the residual metal content of the FL-BP samples was determined by inductively coupled plasma-optical emission spectroscopy (ICP-OES), and the results (Table S1†) show that the amount of any transition metal, which could potentially catalyze the N_2_ activation,^23^ is extremely low, below parts-per million (ppm). However, given the low amount of N_2_ fixed, any contribution of metals in the fixation process cannot be ruled out yet.

The previous experimental studies reported on the adsorption of N_2_ and its further reduction reaction to deliver ammonia (see introduction) were carried out in saturated N_2_ solutions and were driven either by electrochemistry or photoelectrochemistry, and although these reports mainly focused on the obtained ammonia, the XPS results indicated, in both cases, the formation of phosphorus oxide. Nevertheless, due to different experimental reasons, the N1s XPS signal at 402 eV cannot be clearly identified or tracked upon the oxidation that BP suffers.

Quantitatively, XPS shows that a 3 wt% of N atoms are incorporated in the FL-BP sample under ambient conditions after eight weeks (Fig. S7†), however this value must be recalculated with respect to the bulk material since XPS only accounts for species on surface. In order to quantify the real N amount in the whole oxidized FL-BP sample, solid-state magic angle spinning ^31^P nuclear magnetic resonance (ss MAS ^31^P-NMR) measurements were performed. The sample was first measured under strict N_2_ atmosphere in the absence of light (tube wrapped in aluminum foil) and, then, air, light and water were sequentially added (see below). The results (Fig. S9†) show that the oxidation of the sample is only observed under ambient conditions, and the total amount of oxidized P atoms (P–O) is ∼0.1%.^24,25^ Therefore, the total amount of N incorporated in this oxidized FL-BP sample is <0.01%, since the P–O/N ratio is ∼10, according to quantitative XPS.

**Fig. 3:**
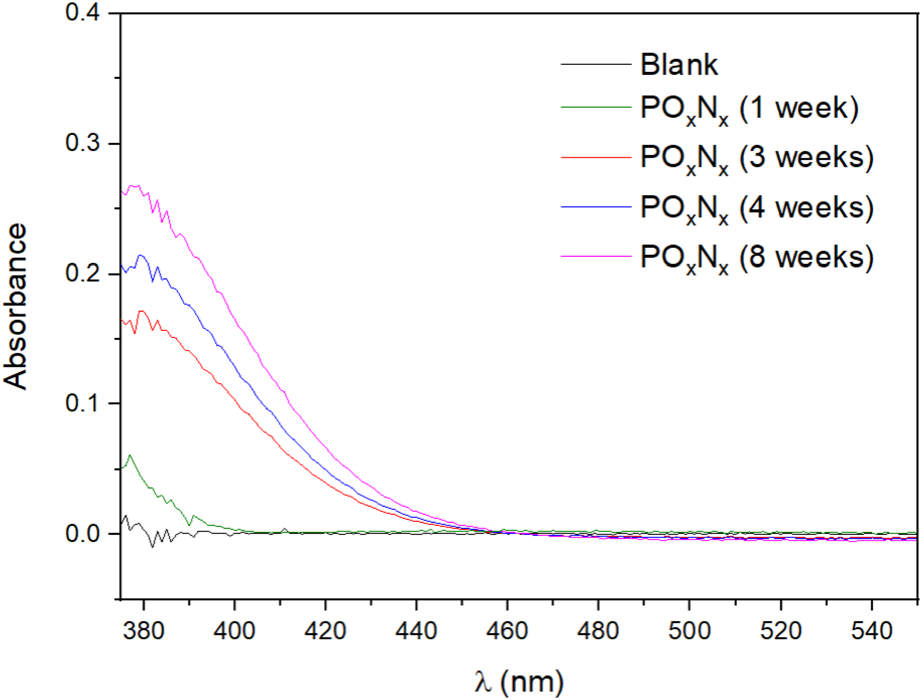
Absorption visible spectrophotometry measurements for the different FL-BP samples exposed to ambient conditions for several weeks and treated by the Nessler protocol to detect NH_3_. Blank refers to a sample of pristine FL-BP.

In order to confirm the estimated <0.01% of N incorporated in the oxidized FL-BP sample, a Nessler test^26^ was performed on different FL-BP samples exposed to ambient conditions, and the results are shown in Fig. 3. The Nessler test shows an increase of NH_3_ on the FL-BP samples with time of exposure to ambient conditions, to reach a plateau at around 8 weeks. A calibration curve gives that the amount of N incorporated into the longest oxidized FL-BP sample is ∼0.01% (Table S2†), in good agreement with the combined XPS/ss NMR results. Thus, one can conclude that, as much, 100 ppm of N_2_ are incorporated in FL-BP after prolonged oxidation under ambient conditions.

Simulated dry air was employed to study the incorporation of N_2_ in FL-BP during the ambient oxidation. When FL-BP was treated with synthetic dry air in a quartz ampule under ambient light for 1 week, neither P oxidation nor N incorporation were observed. If 1 wt% of H_2_O (respect total air) is introduced, and the ampule is wrapped with aluminum foil for 1 week, any change is not observed yet in the material. However, when this mixture is exposed to daylight, P oxidation and N incorporation start to occur. These results in a controlled atmosphere exclude any other N source in the ambient (nitrides, …) rather than N_2_ to be incorporated in the solid material. This result is also in line with the accepted oxidation of FL-BP,^27^ which requires O_2_, H_2_O and light, and strongly supports that the N_2_ fixation process occurs with the oxidation of FL-BP. This combined process has sense from a thermodynamic point of view, since the exothermic formation of new P–O and P=O bonds generate an energy of 335 and 544 kJ mol^−1^, respectively, which would compensate for the energy required to break the N≡N bond (942 kJ mol^−1^). Moreover, one must consider that the chemical oxidation events occur at least 10 times more than the N_2_ breaking, thus providing enough power for the N_2_ breaking to proceed. The N1s XPS signals at ∼402 eV indicate the formation of N–P_*x*_ and N–H_*x*_ bonds and discard the formation of N–O_*x*_ bonds, which should appear below 400 eV. The fact that these N species are mainly on the surface suggests that the 2D structure of FL-BP may participate in the N_2_ fixation process.

**Fig. 4:**
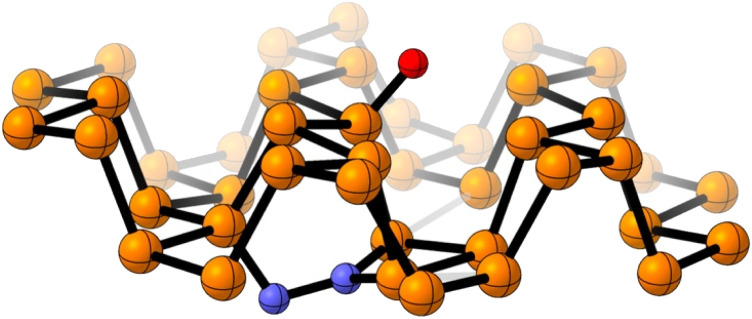
Optimized chemical model for the adsorbed N_2_ on the surface of an oxidized FL-BP model system. Color atom code: orange: phosphorus, red: oxygen, blue: nitrogen.

Computational calculations with Gaussian06 (ref. 28) have been implemented to further support that hypothesis. For the records, the PBE-D3/TZVP^29–31^ density functional theory (DFT) level is used to fully optimized a supercell of 42 phosphorus atoms. That DFT approach was previously used for mimicking phosphorene reactivity.^32^ The performed simulations suggest that N_2_ is very weakly adsorbed on raw FL-BP, and any covalent contact is not observed with the solid surface (Fig. S10†). On the contrary, theory predicts the formation of stable N–P_*x*_ bonds when the oxidized FL-BP is used. As illustrated in Fig. 4, N_2_ is embedded into the phosphorus matrix opposite to the oxidation site. That interaction leads to the activation of the inert N≡N bond, which is elongated from 1.10 Å to 1.40 Å. The performed simulations therefore confirm that the oxidation of phosphorene facilitates the adsorption of N_2_.

In agreement with computed numeric outputs, a ^31^P ss-MAS NMR spectrum, under simulated air, shows signals at 32 and 43 ppm, which could be tentatively assigned to new N containing species, within the reservations associated to the low intensity found (Fig. S9,† bottom). To gain further insights into the possible chemical bonds formed during this oxidative process, we performed Raman spectroscopy measurements on micromechanically exfoliated BP during five weeks. The results indicate an immediate formation of phosphorus-oxygen/nitrogen bonds which disappear after three weeks due to the complete oxidation and degradation of the material. The peaks can be tentatively assigned to P–O–P stretching as well as P–OH and P–N–H bending vibrations (Fig. S11†).^33^ Nevertheless, the formation of such bonds in exfoliated flakes is extremely difficult to trace due to the low concentration that can be expected from these experiments. Further study focusing on the study of the Raman spectra of such chemical species in very well controlled atmospheres and higher nitrogen concentrations would be required to confirm the observed vibrations.

In order to study if this phenomenon is general for elemental P, a sample of just bought and opened red phosphorous (red P) was exposed to ambient conditions and measured by XPS after 8 weeks. The results (Fig. S12†) show that the residual amount of N in the fresh sample increases with time, concomitantly with the oxidation of the red P sample. The Nessler test (Fig. S13†) quantifies the amount of N fixed in red P in 0.003 wt% (30 ppm). This result supports that elemental P fixes N_2_ when oxidized under ambient conditions, regardless of its form, although the particular 2D structure of FL-BP seems to enhance the N_2_ fixation process. Please notice the difficulties associated to quantify these tiny amounts of N in a solid, even the routinely elemental analysis technique has a too low detection level (0.05 wt%).

## 3 Conclusions

We have shown that the 2D material FL-BP reacts not only with O_2_ and H_2_O but also with N_2_ when left under ambient conditions. Indeed, up to 100 ppm of N_2_ can be incorporated in the FL-BP structure after ambient exposure. This process occurs regardless the starting BP form (phosphorene, FL-BP, nanoparticles, flakes…), according to the experimental and computational data here presented, and may have important implications on the chemistry of BP. Besides, red P also suffers this process. Despite much more data are required to unveil the exact underlying mechanism, and given the paramount importance of N_2_ fixation and the abundancy of elemental phosphorous,^34^ this P mediated N_2_ fixation process should be taken into account for future studies on this element and potential catalyzed N_2_ fixing reactions.^35^

## Author contributions

F. G.-P. performed and analyzed the N_2_ fixation reaction. V. L. synthesized the FL-BP samples and carried out the characterization. J. A. V.-M. contributed with the ^31^P MAS-NMR measurements. M. L. and K. J. J. M. contributed in the materials characterization. J. P. C.-C. carried out and interpreted the computational studies. G. A. and A. L.-P. conceived the research, designed the experiments, analysed the data, supervised the project and wrote the manuscript. All the authors discussed the results and contributed to writing the manuscript.

## Conflicts of interest

There are no conflicts to declare.

## Supplementary Material

RA-014-D3RA07331A-s001
