# Supplementary material for: Few-layer black phosphorus enables nitrogen fixation under ambient conditions
Source: RSC Adv. 2024 Feb 5;14(7):4742–7. doi: 10.1039/d3ra07331a (PMC10839751; doi:10.1039/d3ra07331a)
Supplement: RA-014-D3RA07331A-s001 [file RA-014-D3RA07331A-s001.pdf]

Electronic Supplementary Material (ESI) for RSC Advances.
This journal is © The Royal Society of Chemistry 2024

Electronic Supplementary Information

Few-Layer Black Phosphorous Enables Nitrogen Fixation under Ambient Conditions

Francisco Garnes-Portolés,[#,a] Vicent Lloret,[#,b,c] José Alejandro Vidal-Moya,[a] Mario Löffler,[c,d] Karl J. J. Mayrhofer,[c,d] Jose Pedro Cerón-Carrasco,[e] Gonzalo Abellán[*,f] and Antonio Leyva-Pérez.[*,a]

[a] Instituto de Tecnología Química. Universitat Politècnica de València-Consejo Superior de Investigaciones Científicas. Avda. de los Naranjos s/n, 46022, Valencia, Spain. Phone: +34963877800; Fax: +349638 77809.

[b] Department of Chemistry and Pharmacy and Joint Institute of Advanced Materials and Processes (ZMP) Friedrich-Alexander-Universität Erlangen-Nürnberg (FAU) Henkestrasse 42, 91054 Erlangen and Dr.-Mack Strasse 81, 90762 Fürth, Germany. Phone: +49 91165078-65031; Fax: +49 91165078-65015.

[c] Helmholtz-Institute Erlangen-Nürnberg for Renewable Energy (IEK-11), Forschungszentrum Jülich GmbH, Cauerstr. 1, 91058 Erlangen, Germany.

[d] Department of Chemical and Biological Engineering, Friedrich-Alexander University Erlangen-Nürnberg, Cauerstr. 1, 91058 Erlangen, Germany.

[e] Centro Universitario de la Defensa, Academia General del Aire, Universidad Politécnica de Cartagena, C/ Coronel López Peña S/N, Santiago de La Ribera, 30720 Murcia, Spain.

[f] Instituto de Ciencia Molecular (ICMol), Universidad de Valencia, Catedrático José Beltrán 2, 46980, Paterna, Valencia (Spain).

[#] These authors contributed equally.

Corresponding authors: gonzalo.abellan@uv.es, anleyva@itq.upv.es.

Index.

Experimental Section……………………………………………………………..…………....ESI3

Materials and exfoliation process.………………………………………………..…....ESI3

Physical techniques. ……………………………………………………………...ESI3

N_2 fixation process.……………………………………..………………...….ESI4

Nessler test ……………………………………………………………………….ESI4

Computational methods………………………...……...…..……………..……...…ESI5

Tables S1-S2………………………………………………………………..……….….ESI6

Figures S1-S13…………………………………………………………………...…....ESI7

Experimental Section.

- Materials and exfoliation process.

Throughout all experiments, BP with purity higher than 99.999% (Smart Elements) were used. Afterwards, the F.L.-BP flakes were transferred onto Si/SiO_2 substrates (300 nm oxide layer). The exfoliation was performed in argon filled LABmasterpro sp glove box (MBraun) equipped with a gas purifier and solvent vapour removal unit (oxygen and water content lower than 0.1 ppm).

- Physical techniques.

Raman spectra were acquired on a WITec's Raman microscope alpha300 R confocal microscope equipped with an automated XYZ stage. All measurements were conducted using an excitation wavelength of 532 nm. Raman maps were recorded with a laser power of 2 mW, an acquisition time of 2 s, and a grating of 600 grooves/mm. The Raman spectra shown are mean spectra extracted from the recorded Raman maps.

Atomic force microscopy (AFM) was carried out using a Bruker Dimension Icon microscope in tapping-mode. The samples were prepared by spin coating a solution of a given sample at 5.000 rpm. Etalon polysilicon high-resolution tips with a diameter of approximately 10 nm were used to obtain images resolved by 512x512 or 1024x1024 pixels.

Inductively coupled plasma-optical emission spectroscopy (ICP-OES) was determined by a Thermo Scientific ICAP Pro. Solids were disaggregated in concentrated HCl and later diluted in water before analysis.

Absorption spectra were recorded on an Agilent Cary 60 UV-Vis spectrophotometer, in 1 cm wide cuvettes and a xenon source lamp.

X-Ray diffraction spectra were recorded in a CubiX PRO (PAN Analytical) spectrometer, with a Cu K(α) radiation source, 1.5406 Å wavelength.

X-ray photoelectron spectroscopy (XPS) measurements in the ITQ-CSIC/UPV laboratories were performed after sticking the FL-BP material onto a molybdenum plate with scotch tape fil, using a SPECS spectrometer equipped with a Phoibos 150 MCD-9 analyzer using non-monochromatic Mg KR (1253.6 eV) X-ray source working at 50 W. As an internal reference for the peak positions in the XPS spectra, the C1s peak has been set at 284.5 eV. XPS measurements in the HIERN

laboratories were conducted on a Quantera II surface analysis instrument (Physical Electronics Inc., Chanhassen, MN, USA). The powder samples were mounted to the sample holder in a glove box, using double-sided adhesive tape. Under minimization of the exposure to air, the samples were transferred to the XPS vacuum chamber. 100x100 μm^2 homogeneous spots on the samples were irradiated by Al Kα x-rays (1486.6 eV). The samples were neutralized by use of the instrument's neutralizer (Ar^+ and electrons). Photoelectron emission was detected at analyzer pass energies of 112 eV, 55 eV, and 13 eV for detailed elemental spectra of N 1s, C 1s, and P 2p, respectively. All spectra are referenced to the adventitious C 1s binding energy of 284.8 eV.

- ss MAS NMR.

^{31}P MAS NMR spectra were recorded im a Bruker AVIII-HD 400WB. The sample was packed in a 4 mm zicorna rotor and spun at 10 kHz. The spectra were acquired using a recycle delay (d1) of 600 (non oxidized) and 0.5 (oxidized) s, acquiring typically 10000 scans. The sample was first measured under strict N_2 atmosphere in the absence of light (tube wrapped in aluminum foil) and, then, air, light and water were sequentially introduced (each left for one week before measuring). The ^{15}N MAS NMR spectrum was recorded on a 7 mm probe spinning the sample at 5 kHz, using a 6 μs $\pi/2$ and 15 s as recycle delay, with 15000 scans.

- N_2 fixation process.

A sample of solid FL-BP was weighted and left under ambient conditions in the lab for the required time. Alternatively, the sample was placed in a quartz cell, evacuated under vacuum and refilled with synthetic air and/or water, and sealed in capsule form. For the open air samples, 2-3 mg were periodically examined by XPS and UV-vis spectrophotometry (Nessler test). For ss MAS NMR analysis, 200-300 mg of sample were used.

- Nessler test (see Ref. 26 in the main text).

FL-PB (10 mg) and aq. HCl (1 mL, 36 wt%) are allowed to react in a 100 mL volumetric flask for 1 h. Then, three drops of two different stabilizing solutions of MS and PVA are added, and the resulting solution is brought to 100 mL. The Nessler's reagent (1 mL) is added and the mixture is left to react for 5 min. The final mixture is measured by UV-Vis spectrometry, comparing the value

at 400 nm wavelength versus a calibration with 0.0, 0.25, 0.5, 1.0, 1.5 and 3.0 ppm of ammonium chloride in water.

- Computational methods.

The starting geometry was extracted form the Supplemenary Information published by Castriciano, Toffanin and co-workers (Ref. 32 in the main text), which was adapted to builp up a supercell of 42 phosphorus atoms. These molecular models incorporate hydrogen atoms at the boundary sides. The oxidized phosphorene is simulated by incorporating an O^{2-} anion at the central phosphorus atom. Both raw (neutral) and oxidized (doubly negatively charged) phosphorene models are singlets electronic states. To mimick the N_2 adsorption process, a molecule of N_2 is systematically located in the vicinity of all phosphorus atoms in both raw and oxidized models, including up and down faces. The selected DFT level of theory is used to fully optimized to reach a minimum of energy. Frequency calculations confirm that a stable structure is located (no imaginary vibrational modes).

Supplementary Tables.

Table S1. ICP-OES analysis of different metals in the FL-BP material.

Analyte	Concentration average (ppm)
Zn	93.289
Ni	35.367
Co	-4.619
Mn	4.160
Fe	193.288
Mg	184.197
Ca	711.602
Cu	9.740
Al	365.104
Na	4072.274
K	820.338

Table S2. Results for the Nessler test of the different FL-BP materials.

Entry	oxidation time (weeks)	wt% of N
1	1	0.005
2	3	0.061
3	4	0.076
4	8	0.096

Supplementary Figures.

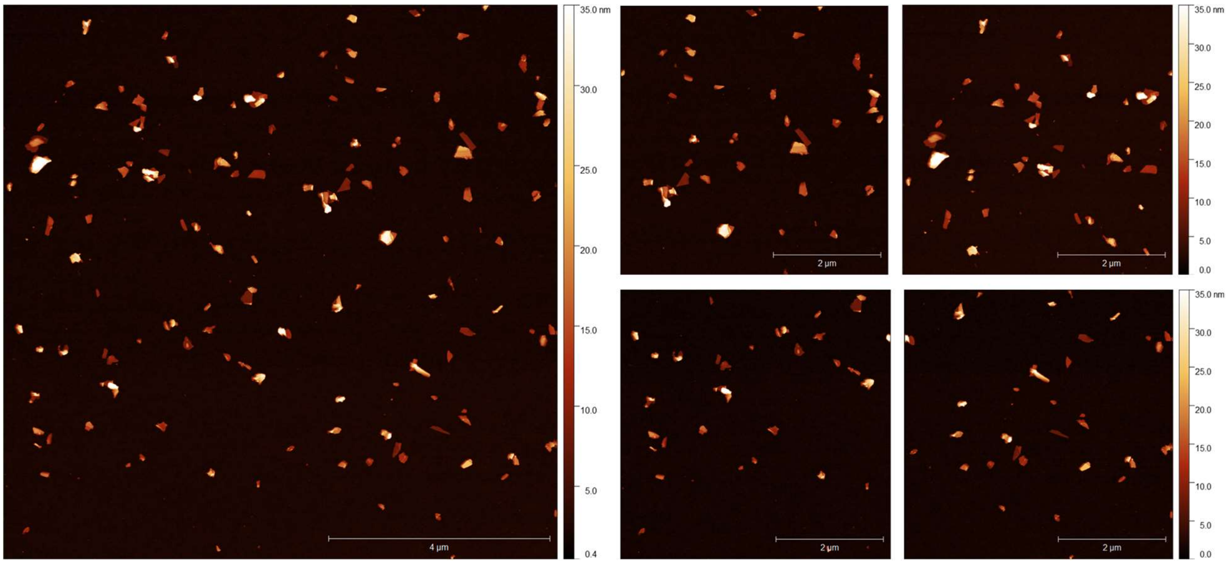

Figure S1. Enlarged AFM image of Figure 1, with four zooms .

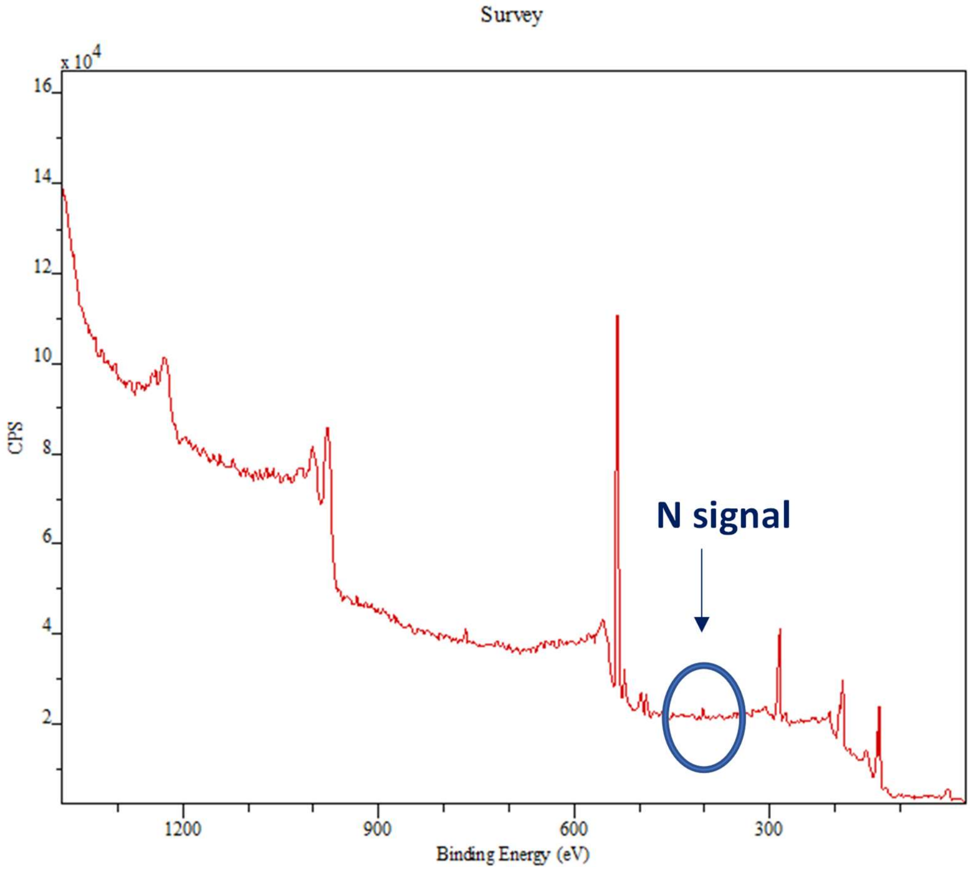

Figure S2. XPS survey spectrum of FL-BP left under ambient conditions for one week in the ITQ-UPV/CSIC laboratory (Spain).

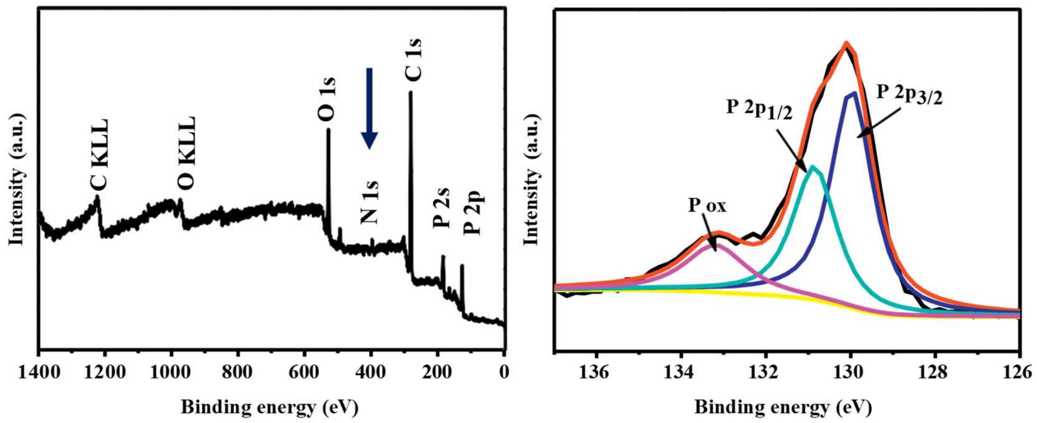

Figure S3. XPS survey and high-resolution P*2p* XPS spectra of holey phosphorene (electrochemically oxidized under air). The blue arrow points to the N signal. Adapted from Ref. 17 in the main text.

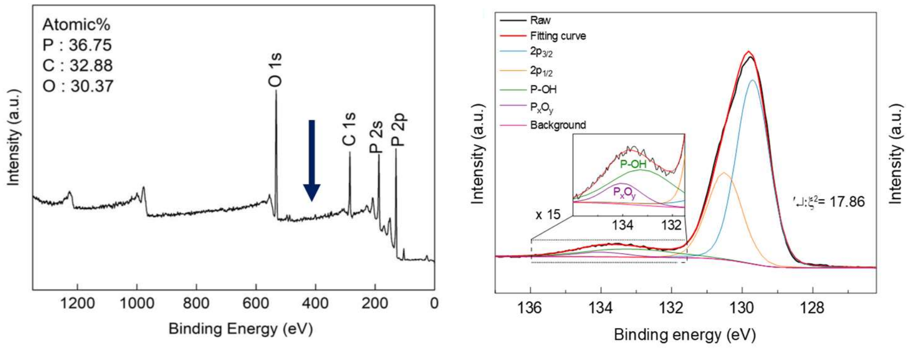

Figure S4. XPS survey (left) and high-resolution P*2p* XPS (right) spectra of oxidized zigzag-phosphorene nanobelts. The blue arrow points to the N signal. Adapted from Ref. 18 in the main text.

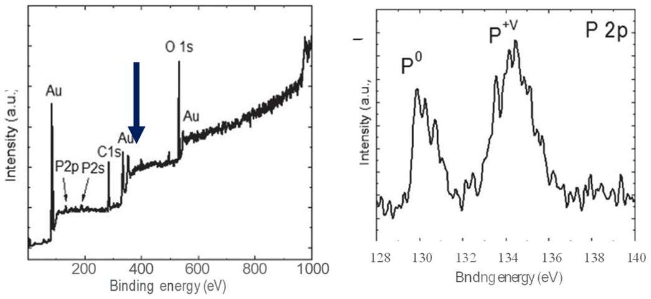

Figure S5. XPS survey (left) and P*2p* XPS (right) spectra of oxidized few-layer black phosphorus nanoparticles. The blue arrow points to the N signal. Adapted from Ref. 19 in the main text.

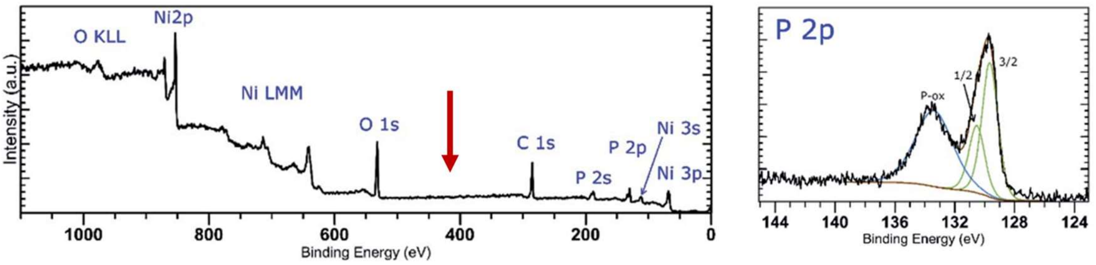

Figure S6. XPS survey and high-resolution P*2p* XPS spectra of Ni-decorated phosphorene. The red arrow denotes the absence of N signal. Adapted from Ref. 20 in the main text.

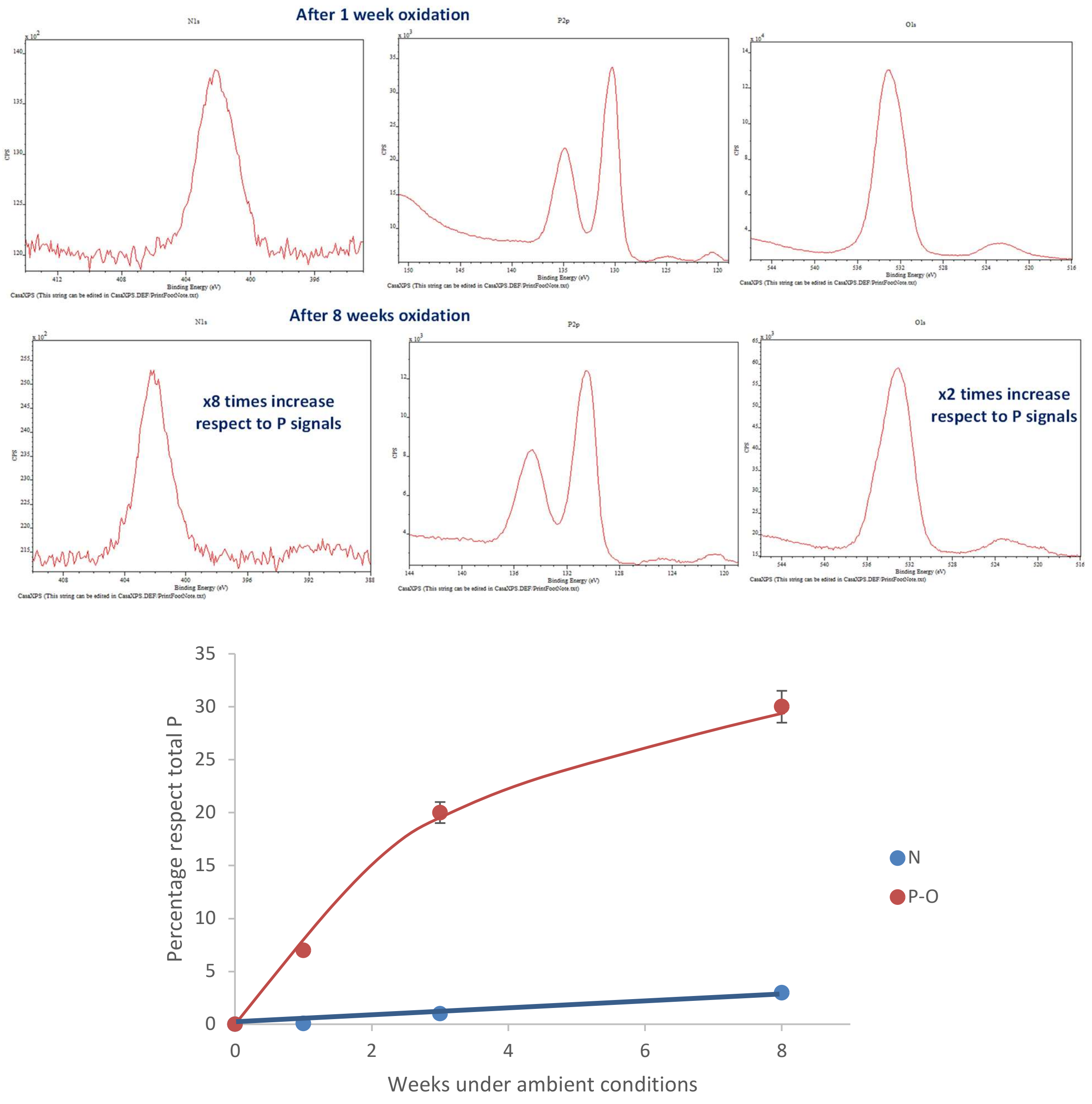

Figure S7. Top: Representative N*1s*, P*2p* and O*1s* XPS spectra of FL-BP left under ambient conditions for 1 week (top) and 8 weeks (bottom). Notice that the peak intensity increases for N and O. Bottom: Correlation between the N incorporation / FL-BP oxidation on surface with time, of the sample under ambient conditions, according to the XPS results. Error bars account for a 5% uncertainty. Lines are a guide to the eye.

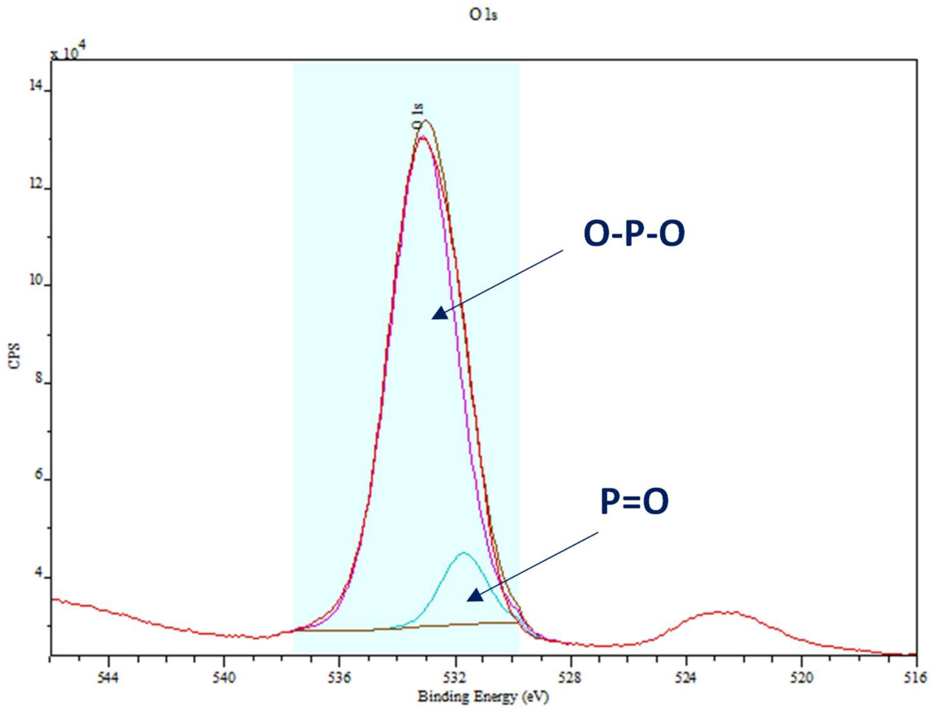

Figure S8. Deconvoluted O*1s* XPS spectrum of FL-BP left under ambient conditions for 8 weeks, managed and recorded in the ITQ-UPV/CSIC laboratory (Spain).

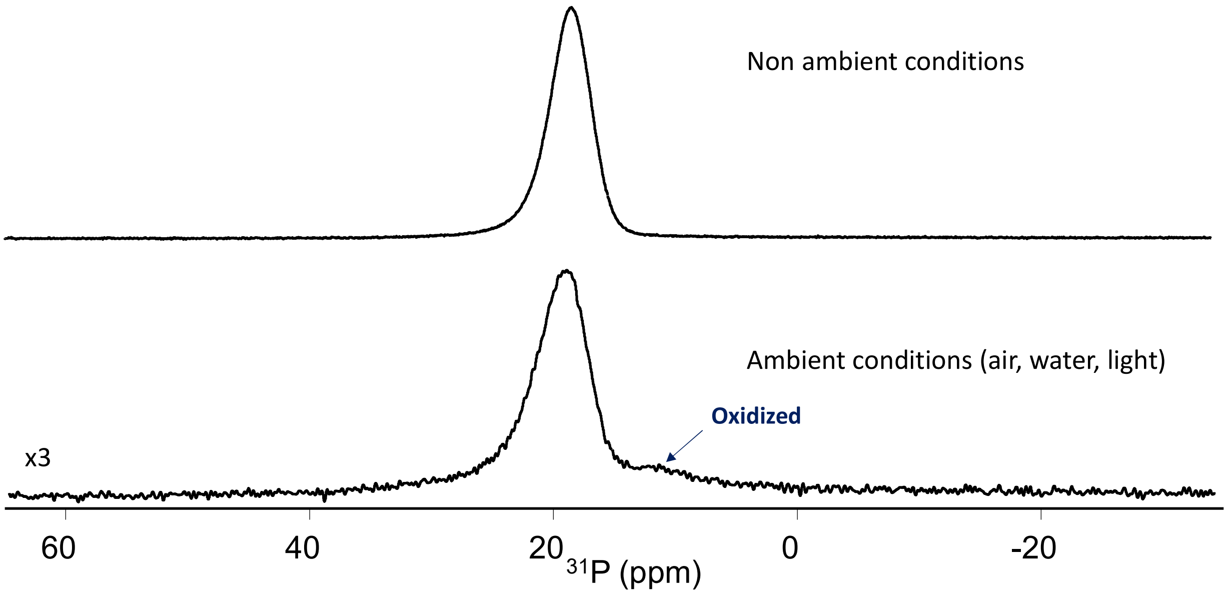

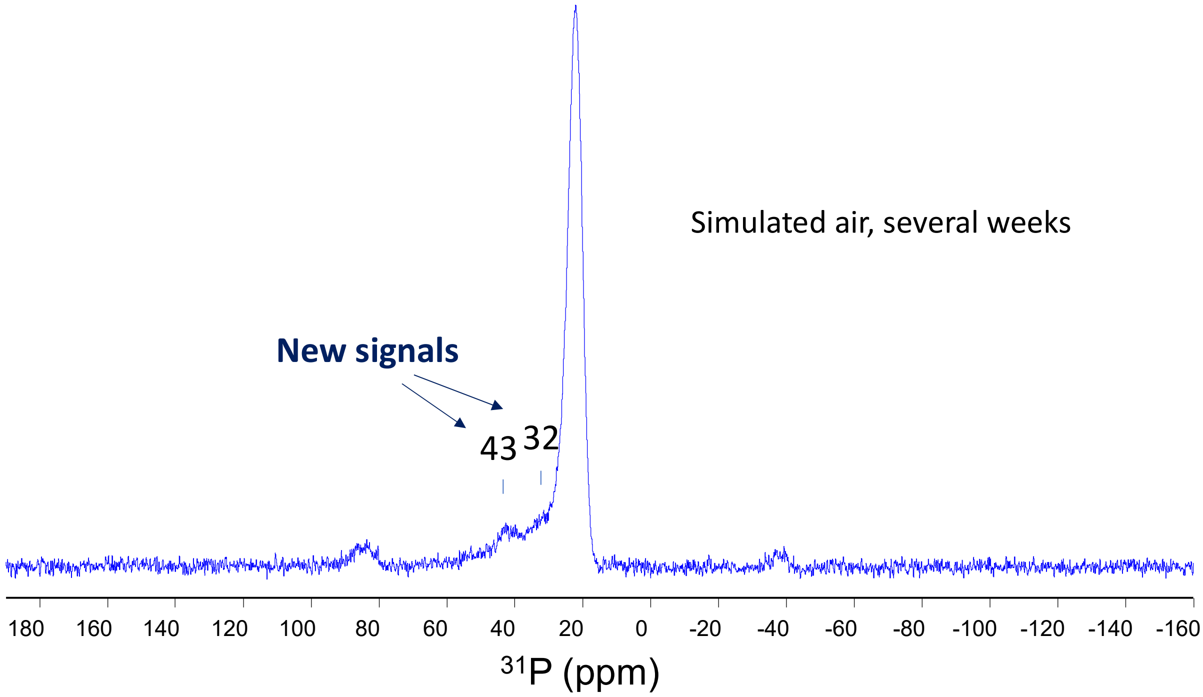

Figure S9. ss MAS ^{31}P-NMR spectra before (top) and after exposure to ambient conditions (middle), and ss MAS ^{31}P-NMR spectra after exposure to simulated ambient air for several weeks (bottom).

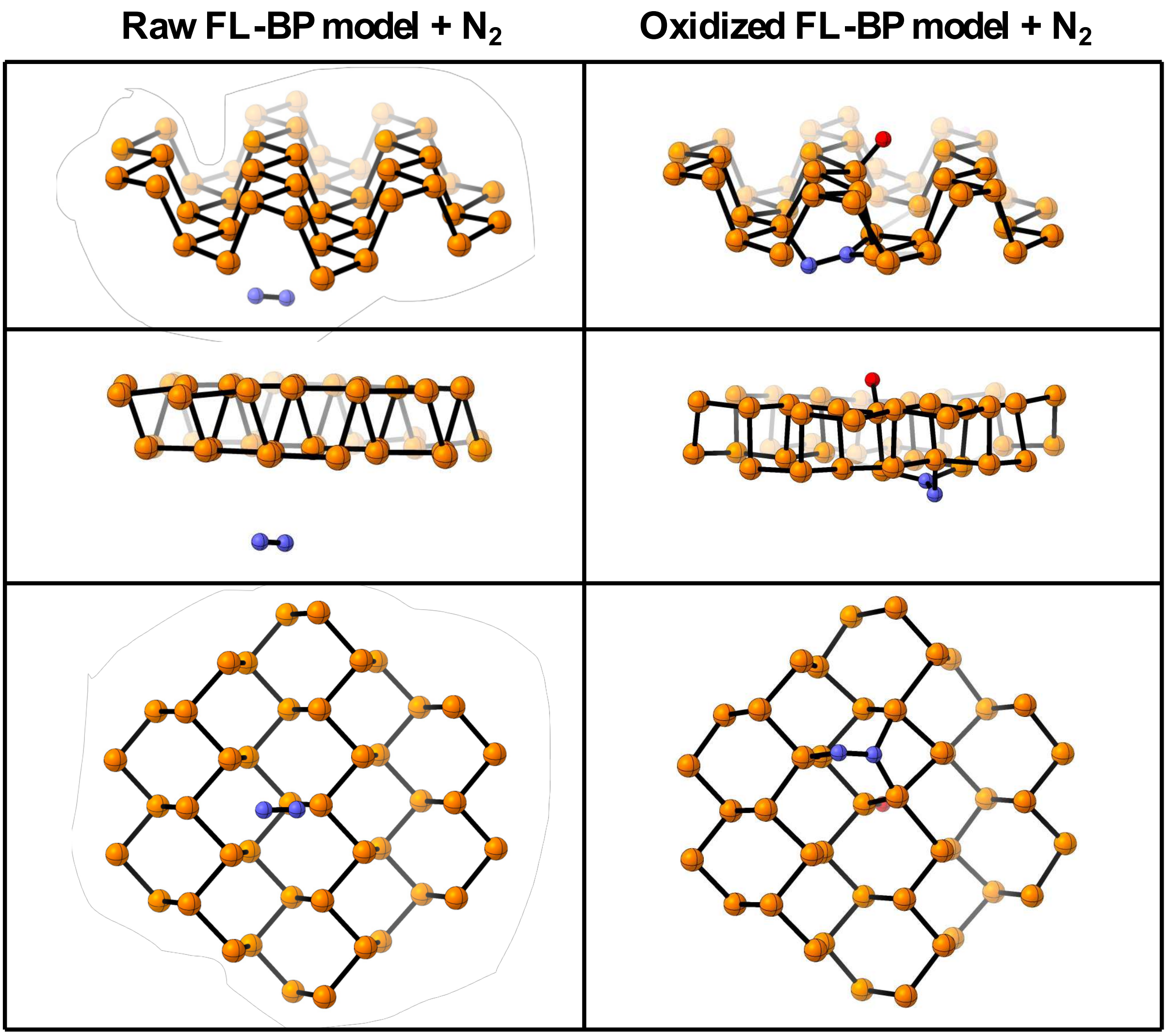

Figure S10. Side and front vies of the fully optimized chemical model for adsorbed N_2 on the surface of the raw (left panel) and oxidized FL-BP (right panel) model systems. Color atom code: orange: phosphorus, red: oxygen, blue: nitrogen. For tha sake of clarity, hydrogen atoms at the boundary sides are not shown. It is remarkable that all attempts to localize stable N-O_x bond failed. The instability of that contact is in agreement with the recorded N*1s* XPS signals.

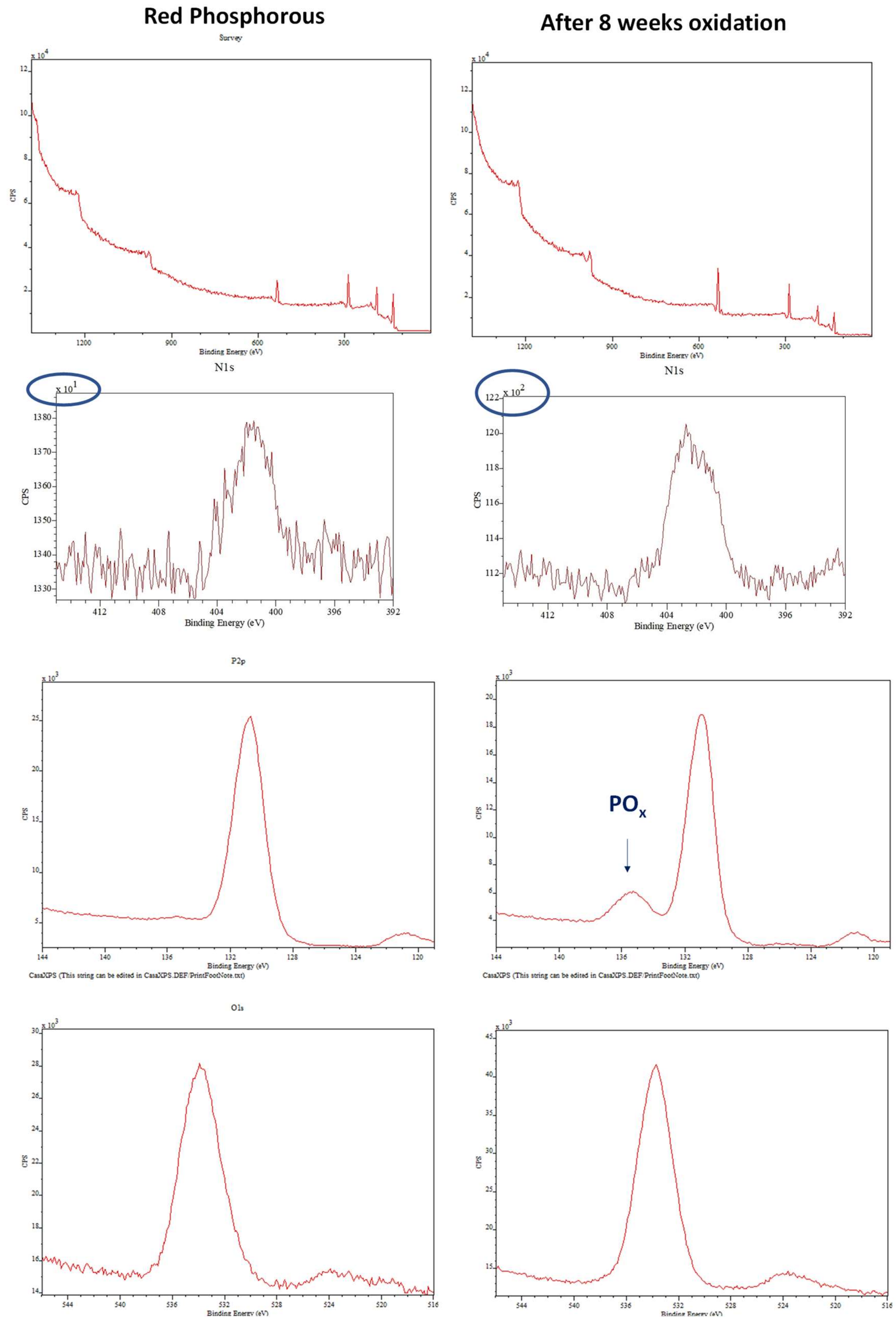

Figure S11. From top to bottom: Survey, N*1s*, P*2p* and O*1s* XPS spectra of untreated red phosporous (left) and left under ambient conditions for 8 weeks (right).

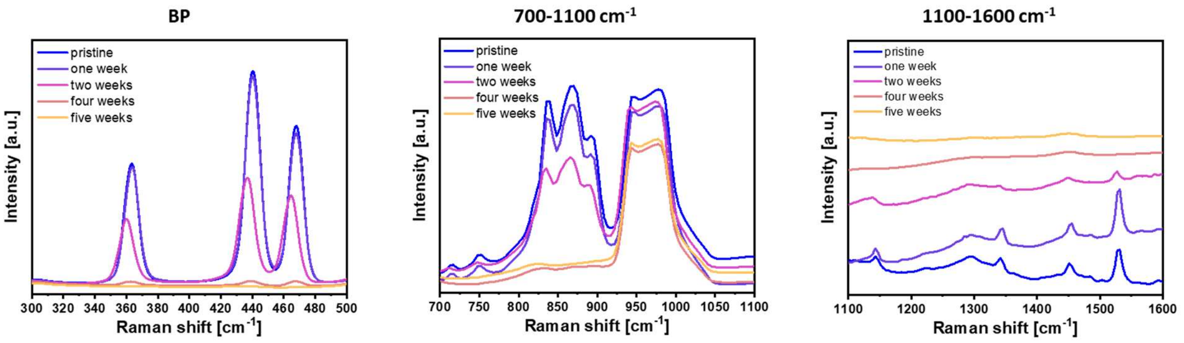

Figure S12. Mean Raman spectra of micromechanically exfoliated BP tracking the formation of phosphorus-oxigen/nitrogen bonds. The peaks at ca. 850 cm^{-1} can be tentatively ascribed to P-O-P stretching and P-OH bending vibrations. The peaks at ca. 1550 cm^{-1} can be tentatively assigned to P-N-H bending virations. Altough the rest of the peaks can be measured for the first weeks and could be asigend to P=O stretching and O-H bending, the signals are even lower in intensity than the previous ones.

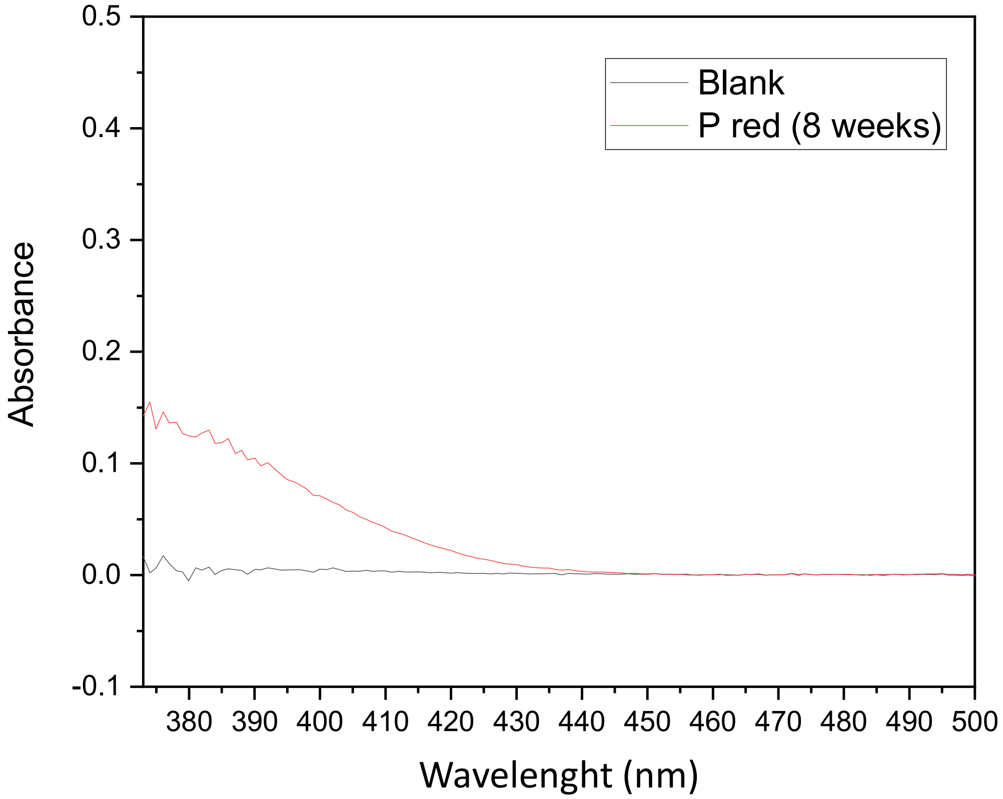

Figure S13. Absorption visible spectrophotometry measurements for a red P sample exposed to ambient conditions for eight weeks and treated by the Nessler protocol to detect NH_3. Blank refers to a sample of pristine red P.
